# Impact of acute cholecystitis comorbidity on prognosis after surgery for gallbladder cancer: a propensity score analysis

**DOI:** 10.1186/s12957-023-03001-0

**Published:** 2023-03-28

**Authors:** Yasuhiro Kihara, Hiroshi Yokomizo, Kenta Murotani

**Affiliations:** 1 Division of General Surgery, Japanese Red Cross Kumamoto Hospital, Kumamoto, 861-8520 Japan; 2 Graduate School of Medicine, Kurume University, Fukuoka, Japan; 3 Biostatistics Center, Kurume University, Fukuoka, Japan

**Keywords:** Acute cholecystitis, Gallbladder cancer, Propensity score, Overall survival, Recurrence-free survival

## Abstract

**Background:**

Cholecystitis can represent a comorbidity during gallbladder cancer surgery; nonetheless, the prognostic impact of acute cholecystitis comorbidity remains unclear. This study aimed to evaluate the impact of acute cholecystitis comorbidity on prognosis after gallbladder cancer surgery, with adjustment for background factors using propensity score analysis.

**Methods:**

A total of 218 patients who underwent gallbladder cancer surgery at our institute between 1986 and 2022 were retrospectively included in the analysis. Patients were divided into two groups according to the presence or absence of acute cholecystitis at the time of surgery. Background factors were adjusted by including intraoperative bile leakage as a covariate in propensity score calculation. Overall survival and recurrence-free survival were compared between the two groups using one-to-one propensity score matching and inverse probability weighting.

**Results:**

Of the 218 patients, 37 had coexisting acute cholecystitis. In one-to-one propensity score matching, the overall survival time in the acute cholecystitis group tended to be shorter than that in the non-acute cholecystitis group, although not significantly (hazard ratio, 2.41; 95% confidence interval, 0.96–6.06). Other analyses using inverse probability weighting showed significantly poor overall survival in the acute cholecystitis group. Regarding recurrence-free survival in propensity score matching, the acute cholecystitis group showed a significantly shorter duration than the non-acute cholecystitis group (hazard ratio, 6.69; 95% confidence interval, 1.46–30.6). The inverse probability weighting-adjusted analysis also indicated a significantly higher risk of recurrence in the acute cholecystitis group.

**Conclusions:**

Acute cholecystitis comorbidity at the time of gallbladder cancer surgery may have a negative impact on gallbladder cancer prognosis.

**Supplementary Information:**

The online version contains supplementary material available at 10.1186/s12957-023-03001-0.

## Background

Because gallbladder cancer (GBC) is asymptomatic in the early stage, this type of malignancy may be detected in an advanced and sometimes inoperable state. In fact, the prognosis of GBC is poor, with a reported 5-year survival rate of 5% [1]. Notably, GBC may be detected incidentally during surgery for cholelithiasis or acute cholecystitis (AC) [2, 3]. In the presence of AC, inflammation can increase the pressure in the gallbladder, resulting in gallbladder wall necrosis due to impaired blood flow, and bile can leak into the abdominal cavity [4]. Surgical manipulation can also damage the gallbladder wall, causing bile spillage (BS), which disperses cancer cell-containing bile into the abdominal cavity. Many reports have shown that if the gallbladder is ruptured by surgical procedure, subsequent recurrence of seeding or port-site recurrence may occur. As such, the prognosis is poor even if the patient undergoes reoperation for curative intent [5–10].

The risk of BS is considered to be increased by AC coexistence. In contrast, changes in the microenvironment due to inflammation are generally known to promote cancer growth and have a negative impact on prognosis [11]. However, it remains unclear whether the prognostic impact of AC on GBC results from BS or the inflammatory microenvironment that promotes cancer development.

This study aimed to evaluate the prognostic impact of AC on GBC after adjusting for patient background and clinicopathological factors, including BS, using propensity score (PS) analysis.

## Methods

### Patients

In this study, we enrolled patients who underwent radical resection of GBC at the Japanese Red Cross Kumamoto Hospital between June 1986 and March 2022. Preoperative and postoperative clinical data were retrospectively collected. This study was conducted in accordance with the principles embodied in the Declaration of Helsinki (as revised in 2013) and was approved by the ethics board of the Japanese Red Cross Kumamoto Hospital (permission no. 515). The requirement for informed consent was waived owing to the retrospective nature of this study. We posted a summary of the trial on our website and asked eligible patients to inform us if they wished to be excluded from the study; none of the participants requested exclusion.

**Fig. 1:**
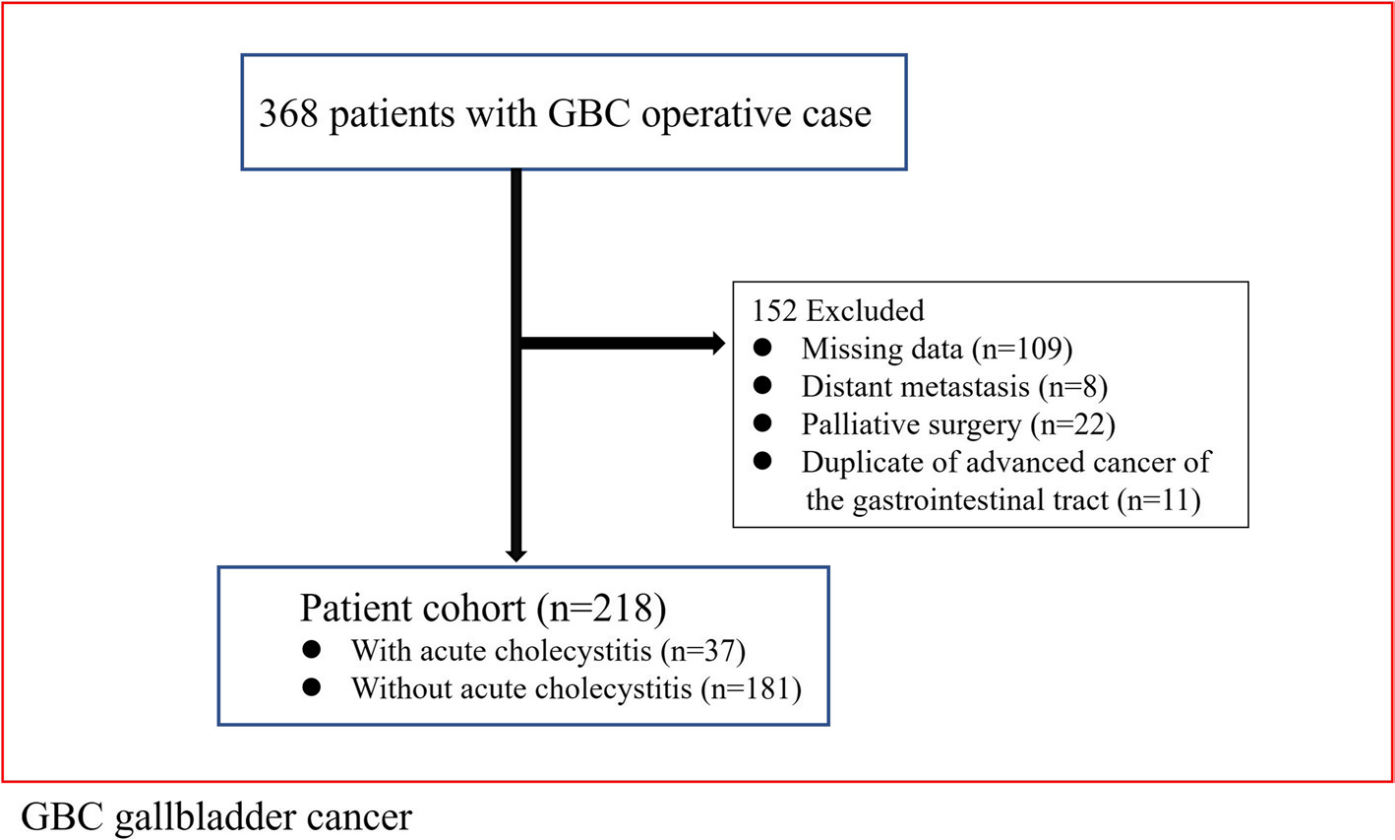
Flow chart of patient enrollment. GBC, gallbladder cancer

We excluded patients from the analysis if they were not able to undergo radical resection due to tumor infiltration and distant metastasis at surgery (22 and 8 patients, respectively). We also excluded 11 duplicate cases with advanced cancer of the gastrointestinal tract. Additionally, 109 cases with missing clinical information, which was required for data analysis in this study, were excluded. Finally, 218 patients were included in the analysis (Fig. 1).

GBC diagnosis was based on the pathology of resected specimens, and staging was classified according to the eighth edition of the Union for International Cancer Control (UICC) staging manual [12].

For cases of so-called incidental GBC in which the patients were operated on for benign gallbladder diseases (e.g., AC, cholelithiasis) and the coexistence of GBC was postoperatively confirmed, a comprehensive decision regarding whether or not additional surgery should be performed was made while taking the degree of cancer progression, the presence of intraoperative BS, age, and performance status into account.

### AC

Preoperative diagnosis of AC was based on physical symptoms (e.g., Murphy’s sign, pain in the right hypochondrium) and systemic inflammatory findings (e.g., fever, elevated white blood cell counts, inflammatory response on blood tests). In addition to these findings, ultrasound and computed tomography imaging studies were conducted to confirm the diagnosis of gallbladder enlargement, wall thickening, and edematous changes in the surrounding area.

The final diagnosis was based on intraoperative findings of gallbladder enlargement, edematous changes around the gallbladder, and abscesses due to wall perforation, as well as gallbladder wall congestion, edema, hemorrhage, and intramural abscess on histopathological examination. For cases in which AC was preoperatively diagnosed, surgical treatment was adopted as the first choice, and drainage procedures (e.g., percutaneous transhepatic gallbladder drainage) were not performed.

### Surgical procedure

For preoperative GBC diagnosis, laparotomy was routinely performed. However, for preoperative diagnosis of gallstones, gallbladder polyps, or cholecystitis, laparoscopic surgery was also performed. Lymph node dissection (LND) was limited to sampling or dissection of pericholedochal lymph nodes at depths up to T1. D2 LND was routinely performed at T2 and above [13]. If surgery was performed for benign disease and GBC was detected postoperatively, D2 LND, resection of the liver bed, or extrahepatic bile duct resection was added to obtain negative resection margins. Alternatively, in some cases, additional port-site resection was performed if BS occurred during the initial surgery. Pre- and postoperative adjuvant chemotherapy was generally not administered.

### Outcome

The primary endpoint of the study was overall survival (OS), and the secondary endpoint was recurrence-free survival (RFS), comparing AC patients with and without concomitant AC after adjusting for background factors. OS was defined as the time from the date of surgery to the last follow-up (in months). RFS was defined as the time from the date of surgery to the date of confirmed recurrence or last follow-up. The 5-year OS and RFS were calculated and used for evaluation.

Additionally, the incidence of recurrent GBC cases was compared according to the presence of AC and BS. Local recurrence was defined as disseminated recurrence in the abdominal cavity near the gallbladder resection site. Distant recurrence was defined as metastatic recurrence in the liver, lung, or distant lymph nodes.

### PS analysis

We used PS matching (PSM) analysis to balance the significant variables of patient background and clinicopathological factors. PSs were calculated using logistic regression analysis, with the probability of AC as the dependent variable and age, sex, American Society of Anesthesiologists physical status (ASA-PS) score, tumor–node–metastasis classification and pathological stage according to the eighth edition of UICC Staging Manual, degree of LND, operative time, blood loss, and intraoperative BS as covariates. The goodness of fit of the logistic model used to estimate the PS was evaluated with a C statistic of 0.883 (95% confidence interval [CI], 0.833–0.934). On the logit of the PS for the presence of cholecystitis, one-to-one matching without replacement was performed using the nearest neighbor match with the caliper width set to 0.20 times the standard deviation (SD) of the logit of the PS. The balance of covariates between groups before and after one-to-one matching was assessed using the *p*-value and standardized difference.

In addition to one-to-one PSM, we compared the outcomes between the two groups using inverse probability weighting (IPW) analysis, stabilized IPW, and truncated (at the 99th percentile) IPW as sensitivity analyses.

### Statistical analyses

Survival curves were estimated using the Kaplan–Meier method, and a comparison between two survival curves was conducted using the log-rank test under one-to-one PSM and adjusted IPW. The Cox proportional hazards model was used to calculate the hazard ratio (HR) for AC for each survival analysis.

Continuous variables were expressed as mean or median with SD or range. Categorical data were analyzed using either the chi-squared test or Fisher’s exact test, whereas continuous data were analyzed using Student’s *t*-test or Wilcoxon’s rank-sum test for unpaired data.

All statistical analyses were performed using JMP® Pro 16.2.0 software (SAS Institute Inc., Cary, NC, USA) and R version 4.0.0 (the R Foundation for Statistical Computing, Vienna, Austria). All analyses were two-tailed, and *p* < 0.05 was considered statistically significant.

## Results

### Patient characteristics

An additional table file shows the clinical characteristics of the 218 patients included in the analysis (Additional file 1). Overall, 49.5% of patients were men, with a median age of 70 years (range, 35–90 years) and mean body mass index of 23.7 kg/m^2^ (*SD*: 3.71, data from 183 out of 218 patients). Among 218 cases, T2 was the most common tumor depth (49%, 106 cases); furthermore, coexisting AC was present in 37 cases (17%), and intraoperative BS occurred in 39 cases (18%). Additionally, 131 patients (60%) were preoperatively diagnosed with having suspected GBC, and 82 patients (38%) had incidental GBC. Laparoscopic surgery was performed in 42 cases (19%) during the initial surgery.

### PS analysis

The distribution of each covariate before and after PSM is shown in Table 1. In the AC and non-AC groups, covariates before adjusting for the PS significantly differed by ASA-PS, degree of LND, intraoperative BS, and operative time. However, these differences were eliminated after adjustment.

**Table 1:** Clinical characteristics of the patients before and after propensity score matching

		**Unmatched comparison**						**Matched comparison**					
		**Without AC (*n*** **= 181)**		**With AC (*n*** **= 37)**		***p***	**SMD**	**Without AC (*n*** **= 25)**		**With AC (*n*** **= 25)**		***p***	**SMD**
**Covariates**	**Category**	***N***	**%**	***N***	**%**			***N***	**%**	***N***	**%**		
Age	< 70	86	48%	17	46%	0.86	0.03	12	48%	12	48%	1.00	< 0.001
	≥ 70	95	52%	20	54%			13	52%	13	52%		
Sex	Male	90	50%	18	49%	0.90	0.02	15	60%	11	44%	0.25	0.32
	Female	91	50%	19	51%			10	40%	14	56%		
ASA-PS	1 or 2	167	92%	29	78%	0.01	0.40	16	64%	18	72%	0.54	0.17
	3 or 4	14	8%	8	22%			9	36%	7	28%		
TNM classification (UICC 8th edition)													
T	0 or 1	76	42%	18	49%	0.23	0.30	8	32%	10	40%	0.47	0.35
	2	92	51%	14	38%			15	60%	11	44%		
	3 or 4	13	7%	5	13%			2	8%	4	16%		
N	0	155	86%	28	76%	0.13	0.08	20	80%	17	68%	0.33	0.28
	≥ 1	26	14%	9	24%			5	20%	8	32%		
M	0	181	100%	37	100%			25	100%	25	100%		
Stage	0 or 1 or 2	149	82%	27	73%	0.19	0.23	20	80%	17	68%	0.33	0.28
	3 or 4	32	18%	10	27%			5	20%	8	32%		
Lymph node dissection	D0	41	23%	17	46%	0.004	0.51	9	36%	10	40%	0.77	0.08
	D1 or D2	140	77%	20	54%			16	64%	15	60%		
Intraoperative bile spillage	Negative	164	91%	15	41%	< 0.001	1.24	15	60%	14	56%	0.77	0.08
	Positive	17	9%	22	59%			10	40%	11	44%		
Intraoperative blood loss	< 200 mL	97	54%	11	30%	0.008	0.50	7	28%	10	40%	0.37	0.26
	≥ 200 mL	84	46%	26	70%			18	72%	15	60%		
Operative time (min)^a^		200 (97)		201 (97)		0.98	0.01	206 (88)		195 (97)		0.66	0.12
Factors not used as covariates in the propensity score matching analysis													
		**Unmatched comparison**						**Matched comparison**					
		**Without AC (*n*** **= 181)**		**With AC (*n*** **= 37)**		***p***	**SMD**	**Without AC (*n*** **= 25)**		**With AC (*n*** **= 25)**		***p***	**SMD**
**Covariates**	**Category**	***N***	**%**	***N***	**%**			***N***	**%**	***N***	**%**		
Laparoscopic surgery	No	148	82%	28	76%	0.39	0.005	22	88%	22	88%	1.0	< 0.001
	Yes	33	18%	9	24%			3	12%	3	12%		
Incidental GBC	Negative	133	73%	3	8%	< 0.001	1.78	11	44%	1	4%	0.001	0.887
	Positive	48	27%	34	92%			14	56%	24	96%		
Preoperative diagnosis of suspected GBC	No	54	30%	33	89%	< 0.001	1.52	13	52%	22	88%	0.012	0.80
	Yes	127	70%	4	11%			12	48%	3	12%		

*AC* acute cholecystitis, *SMD* standardized mean difference, *ASA-PS* American Society of Anesthesiologists physical status, *UICC* Union for International Cancer Control, *GBC* gallbladder carcinoma

^a^Data are presented as mean (standard deviation)

### OS

**Fig. 2:**
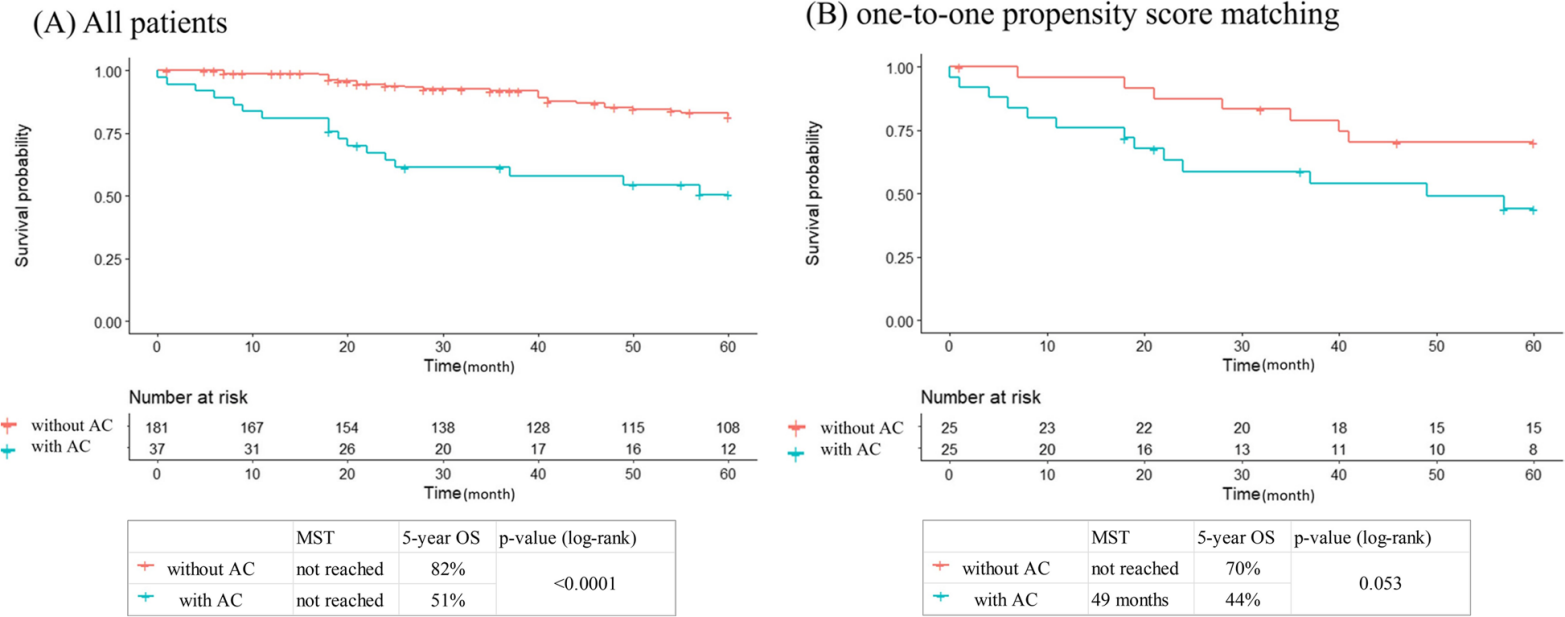
Overall survival. Comparison of overall survival curves after surgery for GBC in patients with AC coexistence and in those without this condition. Comparisons with **a** all patients and **b** one-to-one propensity score matching. GBC, gallbladder cancer; AC, acute cholecystitis; MST, median survival time; OS, overall survival

Among all patients, OS was significantly shorter in the coexisting AC group than that in the non-AC group (*p* < 0.001, log-rank test; Fig. 2a). In one-to-one PSM, the OS in the AC group tended to be shorter than that in the non-AC group, although not significantly (*p* = 0.053, log-rank test; Fig. 2b). In the Cox proportional hazards regression analysis with one-to-one PSM, the risk of death tended to be higher in the AC comorbid group, although not significantly (*HR* = 2.41, 95% *CI*, 0.96–6.06; Table 2). In the IPW-adjusted analysis, each analysis showed a significantly higher risk of death in the AC comorbid group (IPW: *HR* = 3.42, 95% *CI*, 1.63–7.16, stabilized IPW: *HR* = 3.51, 95% *CI*, 1.64–7.51, truncated IPW: *HR* = 3.16, 95% *CI*, 1.48–6.75; Table 2).

**Table 2:** Hazard ratio of acute cholecystitis in the Cox proportional hazards model for OS and RFS

	OS			RFS		
Method	***N***	HR (95% ***CI***)	***p***-value	***N***	HR (95% ***CI***)	***p***-value
Unadjusted	218	3.98 (2.17, 7.32)	< 0.001	218	3.57 (1.80, 7.10)	< 0.001
PSM (one to one)	50	2.41 (0.96, 6.06)	0.061	50	6.69 (1.46, 30.6)	0.014
IPW	380	3.42 (1.63, 7.16)	0.0011	380	3.68 (1.63, 8.30)	< 0.001
Stabilized IPW	218	3.51 (1.64, 7.51)	0.0012	218	3.84 (1.76, 8.37)	< 0.001
Truncated IPW	373	3.16 (1.48, 6.75)	0.0029	373	3.66 (1.62, 8.31)	0.0018

*OS* overall survival, *RFS* recurrence-free survival, *HR* hazard ratio, *CI* confidence interval, *PSM* propensity score matching, *IPW* inverse probability weighting

### RFS

**Fig. 3:**
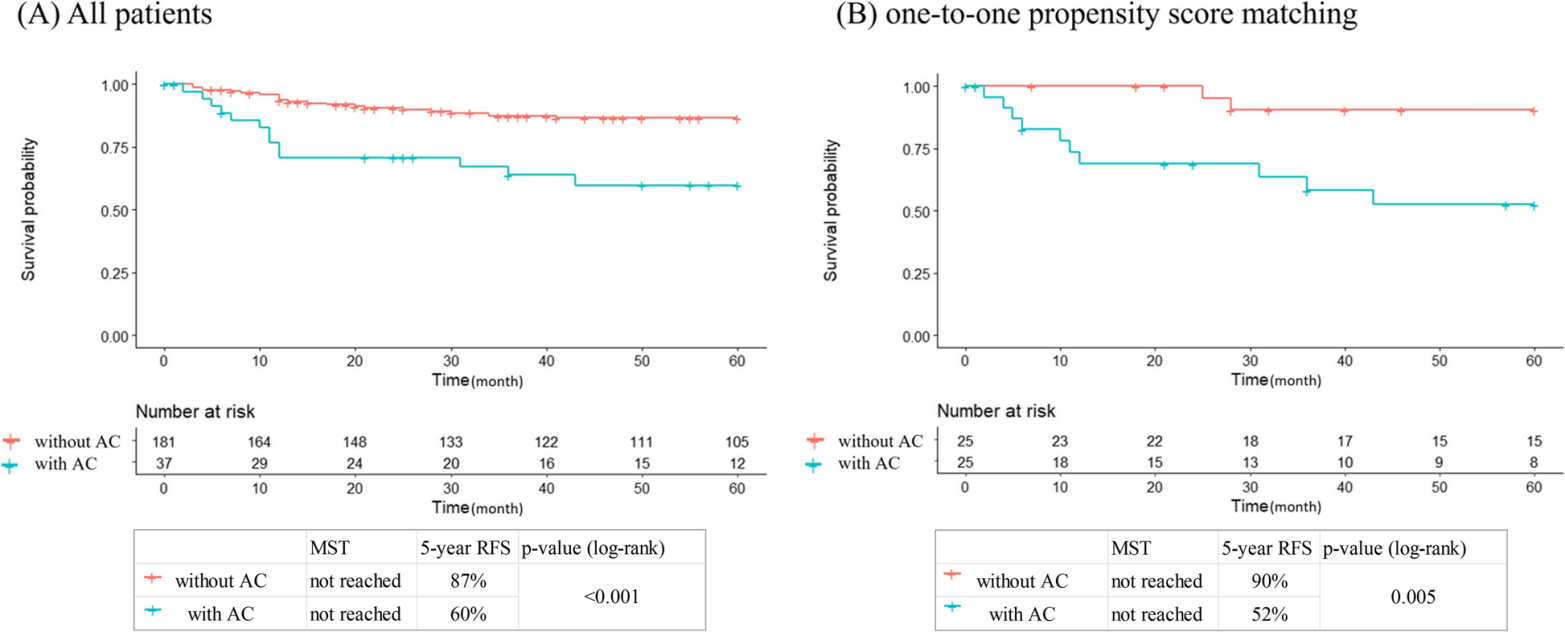
Recurrence-free survival. Comparison of recurrence-free survival curves after surgery for GBC in patients with AC coexistence and in those without this condition. Comparisons with **a** all patients and **b** one-to-one propensity score matching. AC, acute cholecystitis; GBC, gallbladder cancer; MST, median survival time; OS, overall survival

The RFS in all patients and one-to-one PSM was significantly shorter in the AC comorbid group (all patients: *p* < 0.001, Fig. 3a; one-to-one PSM: *p* = 0.005, log-rank test, Fig. 3b). In the Cox proportional hazards model, each analysis revealed that the risk of recurrence was significantly higher in the AC comorbid group (one-to-one PSM: *HR* = 6.69, 95% *CI*, 1.46–30.6, IPW: *HR* = 3.68, 95% *CI*, 1.63–8.30, stabilized IPW: *HR* = 3.84, 95% *CI*, 1.76–8.37, truncated IPW: *HR* = 3.66, 95% *CI*, 1.62–8.31; Table 2).

### Recurrence pattern of GBC with AC

An additional table file shows the results of our analysis of the recurrence pattern of GBC (Additional file 2). GBC recurred in 36 out of 218 patients, and AC and BS were associated with recurrence (*AC*: *p* = 0.0001, *BS*: *p* = 0.008; Table S2A). Given that AC and BS showed a significant association (*p* < 0.0001; Table 1), the subsequent analyses were also stratified according to the presence or absence of BS. The presence of AC in BS-negative cases (*n* = 179) was significantly associated with recurrence (*p* = 0.034; Table S2B). In contrast, in BS-positive cases (*n* = 39), no significant difference in the association between AC and recurrence was identified (*p* = 0.12; Table S2B). Among recurrent GBC cases, local recurrence and distant metastatic recurrence were observed in 20 and 27 patients, respectively; furthermore, duplicate cases were identified in 11 patients. With respect to the local recurrence of GBC, AC was significantly associated with local recurrence in the BS-negative group (*p* = 0.006; Table S2C), but not in the BS-positive group (*p* = 1.0; Table S2B). As for the distant metastatic recurrence of GBC, AC showed no significant association with distant metastatic recurrence in the BS-negative group (*p* = 1.0; Table S2D); conversely, a significant association was observed in the BS-positive group (*p* = 0.045; Table S2D).

## Discussion

In this study, the impact of AC coexistence on long-term prognosis for the surgical treatment of GBC was evaluated by statistical analysis after adjusting for background factors using PS. Several reports previously indicated that intraoperative BS represented a poor prognostic factor [5–10]. In this study, we investigated the prognostic impact of coexisting AC alone on prognosis by PS adjustment for intraoperative BS as one of the background factors. As a result, the AC group had a significantly poorer prognosis in all analyses for both OS and RFS. Furthermore, cases of AC without BS were significantly associated with the local recurrence of GBC.

Chronic inflammation is considered a risk factor for cancer development in various carcinomas [11]. For example, viral hepatitis can lead to chronic liver disease and hepatocarcinogenesis, while *Helicobacter pylori* infection causes chronic gastritis and gastric carcinogenesis [14–17]. In contrast, changes in the local microenvironment caused by acute inflammation may promote the growth and metastasis of preexisting cancers [18]. In general, inflammatory cytokines, such as interleukin (IL)-1, IL-6, and tumor necrosis factor-α, are secreted during the acute phase of inflammation. While these molecules could kill cancer cells via direct and indirect mechanisms, they could also promote cancer growth and metastasis by inducing an inflammatory environment [11, 19–21]. Furthermore, in the local microenvironment of preexisting cancer, tumor cells might recruit inflammatory cells by secreting chemokines, creating favorable conditions for tumor growth [22]. High preoperative neutrophil/lymphocyte (N/L) ratio has been reported as a poor prognostic factor for several carcinomas, including GBC [22]. In the acute phase of inflammation, such as AC, the systemic and local environments are often dominated by neutrophils, resulting in a high N/L ratio. The N/L ratio was not measured in the present study; nevertheless, it is expected that the N/L ratio generally increases with the predominance of neutrophils in the presence of cholecystitis, which is consistent with our finding of poor prognosis in the coexistence of AC in GBC.

During AC, increased pressure in the gallbladder lumen can cause necrosis and perforation of the gallbladder wall due to impaired blood flow, resulting in the dissemination of cancer cell-containing internal fluid to the surrounding peritoneum [4]. This phenomenon is considered a cause of tumor seeding and perforation by surgical manipulation as well as a potential reason for the negative prognostic impact of AC coexistence. The results of this study indicated that cases of AC without BS were significantly associated with the local recurrence of GBC, and that AC might lead to a similar pathology to BS resulting from the increased intra-gallbladder pressure due to inflammation.

In discussing the prognosis of GBC, it is necessary to consider whether it was diagnosed preoperatively or discovered incidentally postoperatively [9, 10]. In this study, as shown in the lower part of Table 2, “Factors not used as covariates in the propensity score matching analysis,” even before and after adjusting for patient background using propensity scores, AC coexistence group had more cases of incidental GBC and significantly fewer cases of preoperatively diagnosed and operated for GBC. Although additional surgery was added in some cases where GBC was found postoperatively, the failure to complete radical surgery for GBC at the initial surgery may also have contributed to the difference in long-term prognosis. The potential background of a high incidence of incidental GBC in the AC coexistence group may also have to be considered as a prognostic factor.

To improve the survival rate after surgery for GBC with poor prognosis, both surgery and postoperative adjuvant chemotherapy are important. In Europe and the USA, the results of the BILCAP trial have established 6-month oral capecitabine as the standard of care for postoperative adjuvant chemotherapy [23]. In Japan, there has been no evidence of adjuvant chemotherapy for GBC. However, the ASCOT trial was initiated in 2013 to confirm whether adjuvant chemotherapy with S-1 prolonged OS in patients with resected biliary tract cancer [24]. The trial results, reported in 2022, showed a prognostic benefit of S-1 [25]. Consistent with the findings from this study, a subgroup analysis showed an improved GBC prognosis [25]. It remains unclear whether postoperative adjuvant chemotherapy improves the prognosis of GBC with AC. However, because this study revealed that the prognosis of the AC group was poorer than that of the non-AC group, GBC with AC might be considered as an indication for aggressive adjuvant therapy.

This study had some limitations. First, it was a single-center retrospective study. Because this study design has the advantage that there were no major differences in surgical technique or perioperative care, the impact of variations in surgical and perioperative management on the findings was minimal. However, a multicenter data analysis is desirable to identify universal events. Second, because PS analysis was only adjusted for measured confounders, unmeasured confounders might exist, and their impact must be considered. In this regard, body mass index and tumor differentiation, which were not included as explanatory variables in the PS analysis as a result of many missing values in our database, might have been unmeasured confounders. Reportedly, a history of smoking and a history of alcohol consumption ≥ 72 g/day among men increase the risk of GBC-related death [26], and these factors may be unmeasured confounders in this study. Because this study included many old cases, it was not possible to investigate smoking and alcohol consumption history of all patients. However, for the 50 patients included in the PSM, we were able to determine whether a history of alcohol consumption and smoking was present for 5 of the 15 patients who died within 5 years after surgery; both habits were not present. It is recommended that these unmeasured confounding factors be included in future studies and that prospective data collection be conducted in a multicenter setting. Although the cause of AC was not described in this study, it can be caused by a variety of factors, including gallbladder stones, choledocholithiasis, and acalculous cholecystitis. It is also known that GBC extending into the lumen of the gallbladder can obstruct the gallbladder neck or the cystic duct deferens and contribute to AC [27]. Prognostic analysis of AC by cause is also an issue for a future study.

Overall, 80% (176/218) of all the study participants and 74% (37/50) of those included in the PSM analysis had stages 0 to 2 disease (Table 1). Therefore, our results regarding OS seemed better than the previously reported data [1, 5–10], which included all disease stages due to the small number of patients with advanced stages.

## Conclusions

This study examined the impact of AC coexistence on prognosis after surgery for GBC using PSs with adjustment for background factors. OS and RFS times were significantly shorter in patients with coexisting AC. The results suggested that AC coexistence might worsen the prognosis of patients with GBC.

## Supplementary Information

**Additional file 1.** Clinical characteristics of patients (n = 218).

**Additional file 2.** Cancer recurrence pattern of patients in GBC with AC.

**Additional file 3.** Raw data set.

## Data Availability

The datasets analyzed in this study are included within the article in additional file 3 and were shared with the approval of the ethics board.

## Abbreviations

AC: Acute cholecystitis
ASA-PS: American Society of Anesthesiologists physical status
BS: Bile spillage
CI: Confidence interval
GBC: Gallbladder cancer
HR: Hazard ratio
IL: Interleukin
IPW: Inverse probability weighting
LND: Lymph node dissection
N/L ratio: Neutrophil/lymphocyte ratio
OS: Overall survival
PS: Propensity score
PSM: Propensity score matching
RFS: Recurrence-free survival
SD: Standard deviation
UICC: Union for International Cancer Control
