# Supplementary material for: Impact of acute cholecystitis comorbidity on prognosis after surgery for gallbladder cancer: a propensity score analysis
Source: World J Surg Oncol. 2023 Mar 28;21:109. doi: 10.1186/s12957-023-03001-0 (PMC10045850; doi:10.1186/s12957-023-03001-0)
Supplement: Supplementary file 1 — Additional file 1. Clinical characteristics of patients (n = 218). [file 12957_2023_3001_MOESM1_ESM.docx]

**Additional file 1 Clinical characteristics of patients (n = 218)**

| Characteristic | | Patients (n) | Percent |
| --- | --- | --- | --- |
| *Patient factors* | |  |  |
| Age, years^a^ | | 70 (35-90) |  |
|  | Interquartile range | 61-78 |  |
| Sex | |  |  |
|  | Male | 108 | 49% |
|  | Female (n=54) | 110 | 51% |
| Body mass index (kg/m^2^)^b^* | | 23.5 (3.7) |  |
|  | Interquartile range | 21.4-25.7 |  |
| ASA-PS | |  |  |
|  | Ⅰ | 48 | 22% |
|  | Ⅱ | 148 | 68% |
|  | Ⅲ | 20 | 9% |
|  | Ⅳ | 2 | 1% |
| TNM classification (UICC 8^th^ edition) | | |  |
|  | Tis | 6 | 3% |
|  | 1 | 88 | 40% |
|  | 2 | 106 | 49% |
|  | 3 | 14 | 6% |
|  | 4 | 4 | 2% |
|  | N0 | 183 | 84% |
|  | 1 | 35 | 36% |
|  | 2 | 0 | 0% |
|  | M0 | 218 | 100% |
|  | Stage 0 | 6 | 3% |
|  | Ⅰ | 87 | 40% |
|  | Ⅱ | 83 | 38% |
|  | ⅢA | 5 | 2% |
|  | ⅢB | 33 | 15% |
|  | ⅣA | 4 | 2% |
| *Operative factors* | |  |  |
| Operative time (min)^b^ | | 200 (97) |  |
| Blood loss (ml)^a^ | | 200 (5-1500) |  |
|  | Interquartile range | 40-374 |  |
| Operative procedure (initial surgery) | | |  |
|  | CC (lap) | 137 (41) | 63% |
|  | CC with LND (lap) | 31 (1) | 14% |
|  | CC with liver bed resection | 27 | 12% |
|  | CC with bile duct resection | 13 | 6% |
|  | CC with segment Ⅳb and V resection | 6 | 3% |
|  | CC with PD | 2 | 1% |
|  | Extended right liver lobectomy | 2 | 1% |
| Additional surgery | | 46 | 21% |
| Intraoperative bile spillage | | 39 | 18% |
| Comorbid AC at time of initial surgery | | 37 | 17% |
| Incidental GBC | | 82 | 38% |
| Preoperative diagnosis of suspected GBC | | 131 | 60% |
| Cancer contained in a very small gallbladder polyp | | 5 | 2% |
| Observation period (month)^a^ | | 62 (0-422) |  |
|  | Interquartile range | 26-120 |  |

^a^ Data was presented as median (range), ^b^ data was presented as mean (SD)

^*^ Data from 183 out of 218 patients

SD, standard deviation; ASA-PS, American Society of Anesthesiologists Physical Status;

UICC, Union for International Cancer Control; CC cholecystectomy; LND lymph node dissection;

lap, laparoscopic; PD, pancreaticoduodenectomy; AC, acute cholecystitis; GBC, gallbladder cancer
