# Supplementary material for: Impact of acute cholecystitis comorbidity on prognosis after surgery for gallbladder cancer: a propensity score analysis
Source: World J Surg Oncol. 2023 Mar 28;21:109. doi: 10.1186/s12957-023-03001-0 (PMC10045850; doi:10.1186/s12957-023-03001-0)
Supplement: Supplementary file 2 — Additional file 2. Cancer recurrence pattern of patients in GBC with AC. [file 12957_2023_3001_MOESM2_ESM.docx]

**Additional file 2** Cancer recurrence pattern of patients in GBC with AC

1. **Recurrence of GBC by AC and BS**

| Covariates | Category | No rec. (n=182) | Rec. (n=36) | *P* |
| --- | --- | --- | --- | --- |
| AC | negative | 159 | 22 | 0.0001 |
|  | positive | 23 | 14 |  |
|  |  |  |  |  |
| BS | negative | 155 | 24 | 0.008 |
|  | positive | 27 | 12 |  |

1. **Recurrence of GBC by AC stratified by presence or absence of BS**

|  | | BS negative (n=179) | | |
| --- | --- | --- | --- | --- |
| Covariates | Category | No rec. (n=155) | Rec. (n=24) | *P* |
| AC | negative | 145 | 19 | 0.034 |
|  | positive | 10 | 5 |  |

|  | | BS positive (n=39) | | |
| --- | --- | --- | --- | --- |
| Covariates | Category | No rec. (n=27) | Rec. (n=12) | *P* |
| AC | negative | 14 | 3 | 0.12 |
|  | positive | 13 | 9 |  |

1. **Local recurrence of GBC by AC** **stratified by presence or absence of BS**

|  | | BS negative (n=24) | | |
| --- | --- | --- | --- | --- |
| Covariates | Category | No rec. (n=14) | Rec. (n=10) | *P* |
| AC | negative | 14 | 5 | 0.006 |
|  | positive | 0 | 5 |  |

|  | | BS positive (n=12) | | |
| --- | --- | --- | --- | --- |
| Covariates | Category | No rec. (n=2) | Rec. (n=10) | *P* |
| AC | negative | 0 | 3 | 1.0 |
|  | positive | 2 | 7 |  |

1. **Distant recurrence of GBC by AC stratified by presence or absence of BS**

|  | | BS negative (n=24) | | |
| --- | --- | --- | --- | --- |
| Covariates | Category | No rec. (n=4) | Rec. (n=20) | *P* |
| AC | negative | 3 | 16 | 1.0 |
|  | positive | 1 | 4 |  |

|  | | BS positive (n=12) | | |
| --- | --- | --- | --- | --- |
| Covariates | Category | No rec. (n=5) | Rec. (n=7) | *P* |
| AC | negative | 3 | 0 | 0.045 |
|  | positive | 2 | 7 |  |

GBC, gallbladder cancer; AC, acute cholecystitis; BS, bile spillage; rec., recurrence;
